# Adrenal Mass and Hypokalaemia: The Zebra Among Horses

**DOI:** 10.7759/cureus.62123

**Published:** 2024-06-11

**Authors:** Zsuzsanna Reti, Laszlo Szabo, Radu M Neagoe, Melinda Kolcsar

**Affiliations:** 1 Department of Endocrinology, Faculty of Medicine, George Emil Palade University of Medicine, Pharmacy, Science, and Technology of Targu Mures, Targu Mures, ROU; 2 2nd Department of Surgery, George Emil Palade University of Medicine, Pharmacy, Science, and Technology of Targu Mures, Targu Mures, ROU; 3 Department of Pharmacology and Clinical Pharmacy, George Emil Palade University of Medicine, Pharmacy, Science, and Technology of Targu Mures, Targu Mures, ROU

**Keywords:** hypokalaemia, electrolyte imbalance, hypertension, adrenal mass, pheochromocytoma

## Abstract

Pheochromocytoma rarely presents with unexplained hypokalaemia, although there are some case reports in the literature. The mechanism behind this could be the increased cellular potassium uptake promoted by beta-2-adrenoreceptor hyperactivation and insulin resistance. We present the case of a 68-year-old hypertensive female patient with a unilateral adrenal mass discovered on angio-CT and typical signs of adrenergic hyperstimulation (hypertensive crisis, headache, and sweating) associated with multiple arrhythmic episodes but with normal plasma and urinary catecholamine levels. During the work-up for hormonal hypersecretion and the cessation of anti-aldosterone medication, the patient presented resistant hypokalaemia. Due to uncorrectable hypokalaemia, we were unable to perform hormonal investigations for primary hyperaldosteronism and referred the patient for laparoscopic adrenalectomy. The histological diagnosis revealed left pheochromocytoma. Postoperatively, the patient experienced rebound hyperkalaemia. In a patient with a unilateral adrenal mass and hypokalaemia, besides primary hyperaldosteronism and adrenocorticotropic hormone-independent hypercortisolism, a possible pheochromocytoma should be ruled out as well by the clinician before surgery.

## Introduction

Adrenal incidentalomas are randomly detected masses of the adrenal glands at radiologic investigations performed for other medical reasons. A low radiodensity (<10 HU at native CT scan) suggests benign lesions like lipid-rich adenoma or myelolipoma [1]. Adrenal incidentalomas associated with hypertension and resistance to therapy, hypokalaemia, headache, and diaphoresis need further hormonal evaluation [2,3]. The recommended screening tool for pheochromocytoma is the measurement of urinary or plasma metanephrines; for endogenous cortisol secretion, the low-dose (1 mg) dexamethasone (DXM) overnight suppression test (ruled out if cortisol is below 1.8 ug/dL) is used [1], and for the suspicion of a mineralocorticoid excess, the plasma aldosterone concentration (PAC) (mg/dL) to plasma renin activity (PRA) (ng/mL/hour) ratio is used, with a value over 20 suggesting primary hyperaldosteronism [2]. Pheochromocytomas rarely cause hypokalaemia; a literature search showed few case presentations or case series [4,5]. The occurrence of hypokalaemia in pheochromocytoma can be attributed to several mechanisms. Excess catecholamines can stimulate beta-2-adrenergic receptors, which increases the activity of the Na+/K+ ATPase pump, driving potassium into cells and thereby reducing extracellular potassium levels. More than that, catecholamines can stimulate renin release, leading to increased production of angiotensin II and subsequent aldosterone secretion, promoting renal excretion of potassium and hypokalaemia [6,7]. Almost confirming these, beta-2 receptor agonists (salbutamol) are effective in resistant hyperkalaemia, and in rare cases, beta blockers can induce hyperkalaemia [8]. On the other hand, the increased glycogenolysis and gluconeogenesis facilitated by catecholamine excess can lead to elevated blood glucose, inducing insulin resistance [9]. In response to hyperglycemia and to compensate for insulin resistance, insulin levels may initially rise, promoting further intracellular potassium shift and potentially leading to hypokalaemia. Beta-2-agonists augment insulin's potassium-lowering effect [10].

## Case presentation

A 63-year-old female patient presented to our Endocrinology Department in November 2020 with hypertensive crises mostly occurring in the second part of the day, headaches, and transpirations. The patient had significant cardiovascular and metabolic comorbidities, including grade 3 arterial hypertension since the age of 34 years (treated with several regimes of antihypertensive agents), atrial fibrillation converted electrically to sinus rhythm, chronic coronary syndrome, right-sided internal carotid occlusion, hyperlipidaemia, type 2 diabetes mellitus, and grade 1 obesity. On presentation, she had uncontrolled hypertension under five classes of antihypertensive drugs (daily doses of 4mg of trandolapril, 360mg of verapamil, 2mg of rilmenidine, 50mg of spironolactone, 20mg of furosemide) with BP values rising to 180/100mmHg. She was also on antithrombotic treatment with rivaroxaban 20mg and acetylsalicylic acid 75mg daily, rosuvastatin 20mg and oral glucose-lowering agent gliquidone 30g per day and supplements of potassium salts (potassium aspartate in an equivalent dose of 200mg potassium per day). Clinically, we observed reddish, considered as semi-active abdominal striae.

**Figure 1:**
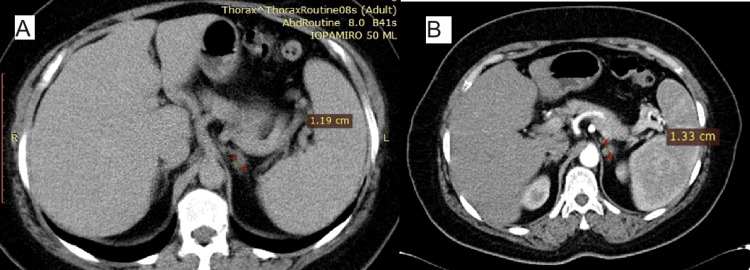
CT scans of the left adrenal mass Arrows showing the adrenal tumour dimensions in consecutive evaluations, year 2010 (Panel A) and year 2011 (Panel B)

Two angio-CT scans performed earlier during cardiologic work-up in 2010 and 2011 described a left adrenal adenoma with a diameter of 11.3 and 13.3 mm, respectively, in consecutive years (Figure 1). Before the presentation, the patient performed investigations for catecholamine excess, which presented normal results. The hormonal panel (see Table 1) completed during hospitalization showed normal thyroid function, slightly elevated basal cortisol level, ACTH concentration approaching the lower limit of the normal range, and elevated late-night salivary cortisol, however, with good cortisol suppressibility, normal urinary free cortisol, no sign of biochemical hyperandrogenism, with normal renal function and serum electrolytes, non-suppressed renin concentration with elevated aldosterone and slightly increased PAC/plasma renin concentration (PRC) ratio but without optimal sampling conditions (aldosterone-antagonist spironolactone and angiotensin/converting enzyme inhibitor not interrupted).

**Table 1:** Hormonal evaluation before surgical intervention * with normal serum K, but on spironolactone HA: hyperaldosteronism; DHEAS: dehydroepiandrosterone sulfate

Parameter	Measured	Normal values	Comment
TSH	1.96 μUI/ml	0.35-4.94	
FT4	0.89 ng/dl	0.7-1.48	
Plasma cortisol – 8 am	19.21 μg/dL	6.01-18.37	
ACTH	8.72 pg/mL	7.2-63.3	
Salivary cortisol – midnight	0.913 μg/dL	<0.41	
Plasma cortisol – after 1 mg DXM suppression test	< 1 μg/dL	< 1,8 ug/dL	
Urinary free cortisol	11.14 μg/24h	1.5-63	
DHEAS	20.5 ug/dl	25.9-460.2	
	0.56 umol/l	0.70-12.49	
Plasma epinephrine	40.2 ng/dL	< 82	
Plasma norepinephrine	435.6 ng/L	80.0-499	
Plasma dopamine	<81.0 ng/L	<93.5	
Urinary epinephrine	<5.34 μug/24h	<27.00	
	0.03 μmol/24h	<0.15	
Urinary norepinephrine	94.47 μg/24h	<97.0	
	0.56 μmol/24h	<0.57	
Urinary dopamine	176.46 μg/24h	<500.00	
	1.15 μmol/24h	<3.26	
Plasma aldosterone	49.300 ng/dL	2.210-35.300	Orthostatic
		1.170-23.60	Clinostatic
Plasma renin* concentration	11.18 μUI/mL	4.4-46.1	Orthostatic
		2.8-39.9	Clinostatic
PAC-PRC-ratio	4.4		>2.4, suggestive of primary HA

The repeated CT scan did not detect an increase in the size of the left adrenal lesion: native scan 8 HU, absolute washout 64% (>60%), and relative washout 54.7% (>40%), suggesting benignity and adenoma-like lesion. After a pause of one and a half years, the patient returned to our unit. At this time, we repeated the Cushing screening with normal results. Osteodensitometry showed low bone mineral density at the lumbar spine (L1-L4 T score T:-1.7 SD). We postponed the re-evaluation of the renin-aldosterone system, recommending the cessation of the mineralocorticoid receptor antagonist (spironolactone) for at least six weeks and the discontinuation of the angiotensin-converting enzyme (ACE) inhibitor (trandolapril) for at least 5-7 days before blood sampling under strict cardiological observation.

At the next presentation, the laboratory detected a low potassium level (2.83 mmol/L, see Table 2) with normal sodium (144 mmol/L), necessitating a delay in hormonal investigations. We introduced oral potassium supplementation (approximately one g/day) without effect, even with a decrease of serum K, prompting an attempt at parenteral supplementation, which was also unsuccessful even with increased intravenous potassium load. Considering the radiological image and the resistant hypokalaemia suggesting primary hyperaldosteronism, we referred the patient to the general surgery unit for laparoscopic adrenalectomy.

**Table 2:** Electrolyte and general laboratory parameters before and after surgery PTH: Parathormone, HbA1c: glycated haemoglobin, GFR: glomerular filtration rate

Parameter	Before surgery (4 to 1 week before intervention)	After surgery (4 to 6 weeks after intervention)	Normal values
Serum potassium	2.76-3.24	5.1-5.7	3.5-5.1 mmol/L
Serum sodium	147-143	140-142	136-145 mmol/L
Corrected calcium	9.6	9.3	8.8-10.3 mg/dl
Albumin	42.3	39.1	32-46 g/L
PTH	143	89.4	10-68pg/ml
HbA1c	6.4	5.4	4.6-5.8%
Creatinine	0.95	0.71	0-1 mg/dL
GFR	59	82.4	90-120 mL/min/1.73 m^2^
Haemoglobin	13.2	10.6	11-15 g/dL

**Figure 2:**
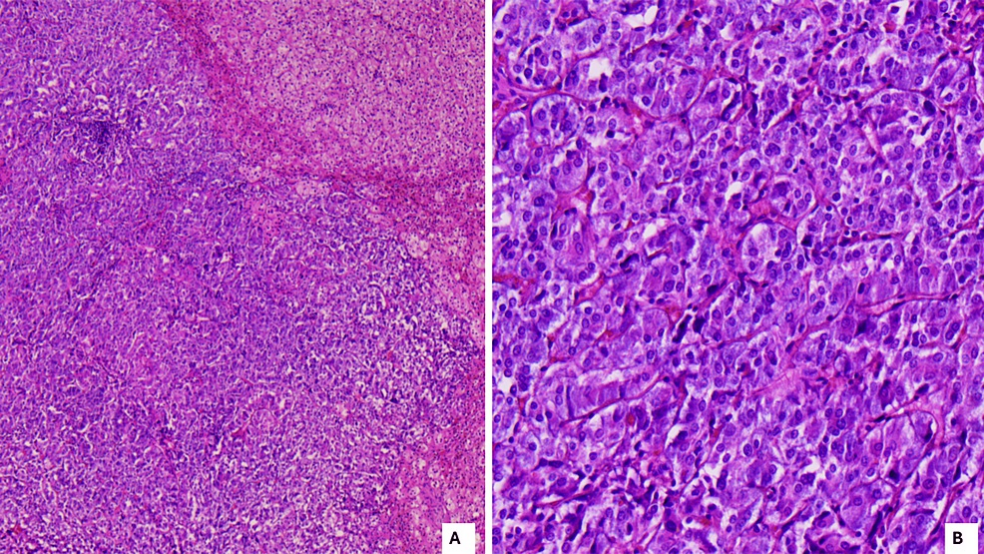
Microscopy of the excised tumour Panel A, microscopy: HE, scale bar 400μm, 5x magnification: tumoural proliferation made of small nests of neuroendocrine cells, poorly delimited from the cortical region. Panel B, microscopy: HE, 100μm, 20x high magnification: Zellballen pattern, with prominent uniform cell nests consisting of polygonal tumour cells with mild pleomorphism. (HE: hematoxylin-eosin staining) (Courtesy of Prof. Dr. Borda A - Pathology Department, Emergency County Hospital/George Emil Palade University of Medicine, Pharmacy, Science, and Technology of Targu Mures)

The pathologist identified a central lesion within the left adrenal gland with a diameter of 10 mm with cells arranged in nests with abundant, finely granulated, dark cytoplasm, uniform nuclei, and moderate vascularisation. Immunohistochemistry revealed positive expression for chromogranin and synaptophysin but negative expression for epithelial membrane antigen and cytokeratin. The final histological diagnosis is left pheochromocytoma (Figure 2).

Following surgery, the patient presented rebound hyperkalaemia (up to 5.7 mmol/L, see Table 2). Consequently, we discontinued the potassium-sparing diuretic and the ACE inhibitor. The favourable glycaemic profile (glycated haemoglobin 5.4%, fasting glucose 97 mg/dL) led us to attribute previous hyperglycaemic episodes to catecholamine hypersecretion (secondary diabetes mellitus) and cease antidiabetic medication (gliquidone). The post-operative adrenocortical insufficiency based on the morning cortisol level (22.4 ug/dL) was ruled out.

The other components of MEN 2B syndrome were also evaluated. The PTH level was moderately elevated (89.4 pg/mL) with normal corrected serum calcium (9.3 mg/dL), suggesting a possible secondary hyperparathyroidism. Thyroid ultrasound and calcitonin measurement (<2 pg/mL) were negative, indicating no evidence of medullary thyroid carcinoma. Urinary metanephrine (71.78 ug/24h) and normetanephrine (253.76 ug/24h) concentrations were within the normal range, and chromogranin A presented normal values. Molecular genetic testing has not yet been performed, but it is planned as recommended in all cases of pheochromocytomas/paragangliomas. 

The follow-up CT scan revealed a hematoma in the surgical field (64x45x66 mm), accompanied by elevated inflammatory markers: erythrocyte sedimentation rate, C-reactive protein, and fibrinogen. Unfortunately, the clinical evolution was unfavourable, necessitating reintervention for drainage of the abscess. Microbiological examination isolated Pseudomonas aeruginosa. One month later, the CT exam still showed a persistent abscess, albeit in reduced size (26x38x36 mm). Following recommendations from the infectious disease colleague, we initiated antibiotic therapy with extended-spectrum coverage (intravenous ceftazidime initially in 3x2 g dose, later switched to oral ciprofloxacin 2x500 mg in dose). Several months later, the patient underwent another surgical drainage for the persistent local infection with a good outcome.

In the last visit, one year after surgery, she presented with hypertension but was well-controlled on medication (calcium-channel blocker and beta-blocker), with no alteration of glucose metabolism.

## Discussion

Both hypokalaemia and catecholamine excess represent an elevated risk for atrial fibrillation [9]. Type 2 diabetes, impaired glucose tolerance and insulin resistance are frequent consequences of pheochromocytomas, and they are usually reversible after tumour removal [11].

In investigating an adrenal mass associated with hypertension, there are well-established recommendations for evaluating hormone hypersecretion. In case of suspicion for endogenous cortisol hypersecretion, guidelines recommend the following first-line screening tests: 24-hour free urinary cortisol, bedtime salivary cortisol and suppression test with 1 mg DXM. Our patient presented only one positive test result (elevated salivary cortisol), which on repeated measurement was normal and ruled out the diagnosis of endogenous hypercortisolism. For the diagnosis, at least two equivocal positive tests are necessary [12].

The ratio between PAC and PRA or PRC should be used as the first line for detecting mineralocorticoid excess. As a non-selective mineralocorticoid receptor antagonist, spironolactone stimulates renin and aldosterone secretion [13]. ACE inhibitors increase renin concentration and lower aldosterone concentration, potentially affecting the accuracy of renin-aldosterone measurements [14]. The generally accepted cut-off for the PAC-PRA ratio is >20 ng/mL/h/ng/dL; alternatively, it is >2.4 mU/L/ng/dL for the PAC-PRC ratio. Both K-sparing diuretics and ACE inhibitors reduce the aldosterone-renin-ratio (ARR). Surprisingly, our patients exhibited an elevated ARR despite concurrent spironolactone and trandolapril usage. This anomaly underscores the importance of plasma renin activity as a more valuable tool for detecting primary hyperaldosteronism than PRC, as utilised in our patient's case [2,15].

For pheochromocytoma screening, measuring 24-hour urinary fractionated metanephrines and catecholamines and plasma fractionated metanephrines (after 30 min in the supine position) is recommended. Plasma catecholamines have a short half-life and, therefore, are considered less reliable (normal range in the case of our patient) [16]. The normal values of serum and catecholamine determinations distracted us from the diagnosis of adrenergic excess preoperatively. Also, the repeated report of an adenoma on radiologic findings and the treatment-refractory hypokalaemia revealed instead a high suspicion of mineralocorticoid excess. In the setting of a benign-looking adrenal lesion (as described several times in our patient by the radiologists) repeating the catecholamine metabolites measurement was not considered necessary. 

Benign adrenal lesions typically present with low density (negative or under 10HU) and demonstrate rapid contrast enhancement and quick contrast washout. On the other hand, malignant lesions enhance rapidly but demonstrate a more delayed washout. The 2023 European Guideline for adrenal incidentalomas recommends a cut-off of 60% for absolute washout (our patient had 64%). Regarding relative washout, the cut-off is less clear: 40% according to the 2020 Guideline, but the most recent Guideline suggests a new, potential, higher limit - 58%. (Our patient's relative washout fell within the grey area - 54.7%) [1,17]. The repeated adrenal imagistic investigations also maintained the description of a lipid-rich adrenal mass, suggesting adenoma instead of the typical image of pheochromocytoma. The mentioned guidelines also recommend against pheochromocytoma screening in the case of a negative HU lesion of the adrenal mass, so our patient did not have the recommendation of repeating catecholamine metabolites due to low suspicion.

In pheochromocytomas, even histological evaluation may not definitively confirm the benign or malign nature of the tumour. However, a low Ki67% index (<1%) and a small tumour size (1 cm) are predictive of a benign pheochromocytoma. The presence or absence of metastasis remains the most critical parameter. Fortunately, our patient has no detected metastasis so far [18].

Our patient had a slightly decreased dehydroepiandrosterone sulfate (DHEAS) level. Accompanied by low-normal ACTH, the possibility of autonomous cortisol secretion arose, but the screening tests ruled out endogenous hypercortisolism. The relationship between DHEAS and chromocytes has been actively researched recently, particularly in the preclinical field. In vitro models have shown that DHEAS decreases the chromocytes' NGF (neuronal growth factor)-induced proliferation and survival. However, more than the available data is needed to draw definitive conclusions [19].

Regarding the association of hypokalaemia in pheochromocytoma, we searched the literature for similar cases. A retrospective study in New York identified a negative significant correlation between plasma epinephrine and potassium concentration in 16 patients with pheochromocytoma. Epinephrine has a more significant effect on plasma potassium concentration compared to norepinephrine. The study found no significant correlation between plasma norepinephrine and potassium levels [4]. The explanation of hypokalaemia could be based on the physiologic action of epinephrine potassium changes. Administration of epinephrine in healthy individuals rapidly decreases serum potassium levels. Nonetheless, the overall potassium loss does not appear significant; instead, it reflects a redistribution of potassium between the extracellular and intracellular space. Propranolol administration (a non-selective beta blocker) prevents this effect. It is well known that adrenaline has a more potent effect on beta-2 adrenergic receptors than noradrenaline [4].

The literature describes rebound hyperkalaemia following adrenalectomy for aldosteronoma, but we were unable to find similar cases after adrenalectomy performed for pheochromocytoma [20].

## Conclusions

Pheochromocytoma, through increased sympathetic activity, predisposes to glycaemic dysregulation, increased risk for arrhythmia, and elevated cardiovascular mortality. In rare cases, hypokalaemia could be a significant consequence of epinephrine hypersecretion, exacerbating cardiovascular morbidity. Notably, rebound hyperkalaemia following adrenalectomy for pheochromocytoma (in the absence of postoperative adrenocortical insufficiency) has not been reported to date.
